# Scanning sequences after Gibbs sampling to find multiple occurrences of functional elements

**DOI:** 10.1186/1471-2105-7-408

**Published:** 2006-09-08

**Authors:** Kannan Tharakaraman, Leonardo Mariño-Ramírez, Sergey L Sheetlin, David Landsman, John L Spouge

**Affiliations:** 1 Computational Biology Branch, National Center for Biotechnology Information National Library of Medicine, National Institutes of Health, 8600 Rockville Pike, MSC 6075 Bethesda, MD 20894-6075, USA

## Abstract

**Background:**

Many DNA regulatory elements occur as multiple instances within a target promoter. Gibbs sampling programs for finding DNA regulatory elements *de novo *can be prohibitively slow in locating all instances of such an element in a sequence set.

**Results:**

We describe an improvement to the A-GLAM computer program, which predicts regulatory elements within DNA sequences with Gibbs sampling. The improvement adds an optional "scanning step" after Gibbs sampling. Gibbs sampling produces a position specific scoring matrix (PSSM). The new scanning step resembles an iterative PSI-BLAST search based on the PSSM. First, it assigns an "individual score" to each subsequence of appropriate length within the input sequences using the initial PSSM. Second, it computes an E-value from each individual score, to assess the agreement between the corresponding subsequence and the PSSM. Third, it permits subsequences with E-values falling below a threshold to contribute to the underlying PSSM, which is then updated using the Bayesian calculus. A-GLAM iterates its scanning step to convergence, at which point no new subsequences contribute to the PSSM. After convergence, A-GLAM reports predicted regulatory elements within each sequence in order of increasing E-values, so users have a statistical evaluation of the predicted elements in a convenient presentation. Thus, although the Gibbs sampling step in A-GLAM finds at most one regulatory element per input sequence, the scanning step can now rapidly locate further instances of the element in each sequence.

**Conclusion:**

Datasets from experiments determining the binding sites of transcription factors were used to evaluate the improvement to A-GLAM. Typically, the datasets included several sequences containing multiple instances of a regulatory motif. The improvements to A-GLAM permitted it to predict the multiple instances.

## Background

Regulation of gene transcription is complex and often combinatorial in nature [1-3]. Combinatorial gene regulation is a major factor in evolution, because it helps coordinate diverse novel phenotypic features in a new species. Because it often reflects chemical synergies between transcription factors (TFs), combinatorial gene regulation can be broadly classified as either: (1) *homotypic*, where a single TF binds to multiple sites in the regulatory region of a gene; or (2) *heterotypic*, where multiple TFs target a single gene. Accurate knowledge of potential synergies between regulatory elements is therefore essential to understanding evolution and phenotypic diversity.

Many computational tools are available for prediction of regulatory elements. Most tools are based on one of two methods: (1) an enumeration of over-represented words [4-9]; or (2) probabilistic sequence models [10-15]. Our previous work [16] produced the A-GLAM computer program, which combines word enumeration with probabilistic sequence models to identify *cis*-regulatory sequences in human promoters, as follows. Given any gapless subsequence alignment, probabilistic sequence models yield a marginal Bayesian log-odds score. The Gibbs sampler in A-GLAM uses simulated annealing to maximize the log-odds score over all possible gapless alignments. A-GLAM also can start from a set of "seeds", e.g., statistically significant positions from word enumeration, to maximize the log-odds score over all possible gapless alignments containing the seeds.

Gibbs sampling (or more descriptively, successive substitution sampling) is a respected Markov-chain Monte Carlo procedure for discovering sequence motifs. As a theoretical framework, however, it encounters several practical problems when searching for regulatory elements in DNA. First, it tends to find DNA repeat elements, regardless of their biological interest. Second, it often requires prohibitive computational time to find multiple instances of a regulatory element in a single sequence.

Because A-GLAM was based on Gibbs sampling, we were eager to overcome the practical problems above. Our previous work [16] used seeds to overcome the first problem, repeats. The user can constrain the alignment output to include the seeds, a so-called "anchored alignment". Our implementation of Gibbs sampling therefore avoids repeats, because the user can specify in advance which motif is of biological interest.

To overcome the second problem, multiple instances of a motif, A-GLAM now has an option for post-processing the results of Gibbs sampling. Gibbs sampling produces a position specific scoring matrix (PSSM). The new scanning step resembles a PSI-BLAST search based on the PSSM. The Methods section describes it under the sub-heading "The new scanning step".

## Implementation

A-GLAM was written in C++ and compiled by gcc (GCC version 3.4.0) under the linux operating environment. The binary files, documentation and the datasets are available for download from the project ftp site [see Additional files 1 &2].

### The Gibbs sampling step in the previous implementation of A-GLAM

Briefly, A-GLAM takes a set of sequences as input. The Gibbs sampler step in A-GLAM uses simulated annealing to maximize an "overall score", a figure of merit corresponding to a Bayesian marginal log-odds score. The overall score is given by

s=∑i=1w(log⁡2(a−1)!(c+a−1)!+∑(j){log⁡2[(cij+aj−1)!(aj−1)!]−cijlog⁡2pj})     (1)
 MathType@MTEF@5@5@+=feaafiart1ev1aaatCvAUfKttLearuWrP9MDH5MBPbIqV92AaeXatLxBI9gBaebbnrfifHhDYfgasaacH8akY=wiFfYdH8Gipec8Eeeu0xXdbba9frFj0=OqFfea0dXdd9vqai=hGuQ8kuc9pgc9s8qqaq=dirpe0xb9q8qiLsFr0=vr0=vr0dc8meaabaqaciaacaGaaeqabaqabeGadaaakeaacqWGZbWCcqGH9aqpdaaeWbqaamaabmGabaGagiiBaWMaei4Ba8Maei4zaC2aaSbaaSqaaiabikdaYaqabaGcdaWcaaqaamaabmGabaGaemyyaeMaeyOeI0IaeGymaedacaGLOaGaayzkaaGaeiyiaecabaWaaeWaceaacqWGJbWycqGHRaWkcqWGHbqycqGHsislcqaIXaqmaiaawIcacaGLPaaacqGGHaqiaaGaey4kaSYaaabuaeaadaGadeqaaiGbcYgaSjabc+gaVjabcEgaNnaaBaaaleaacqaIYaGmaeqaaOWaamWaceaadaWcaaqaamaabmGabaGaem4yam2aaSbaaSqaaiabdMgaPjabdQgaQbqabaGccqGHRaWkcqWGHbqydaWgaaWcbaGaemOAaOgabeaakiabgkHiTiabigdaXaGaayjkaiaawMcaaiabcgcaHaqaamaabmGabaGaemyyae2aaSbaaSqaaiabdQgaQbqabaGccqGHsislcqaIXaqmaiaawIcacaGLPaaacqGGHaqiaaaacaGLBbGaayzxaaGaeyOeI0Iaem4yam2aaSbaaSqaaiabdMgaPjabdQgaQbqabaGccyGGSbaBcqGGVbWBcqGGNbWzdaWgaaWcbaGaeGOmaidabeaakiabdchaWnaaBaaaleaacqWGQbGAaeqaaaGccaGL7bGaayzFaaaaleaadaqadiqaaiabdQgaQbGaayjkaiaawMcaaaqab0GaeyyeIuoaaOGaayjkaiaawMcaaaWcbaGaemyAaKMaeyypa0JaeGymaedabaGaem4DaChaniabggHiLdGccaWLjaGaaCzcamaabmGabaGaeGymaedacaGLOaGaayzkaaaaaa@7E1E@

In Equation (1), *m*! = *m*(*m *- 1)...1 denotes a factorial; *a*_*j*_, the pseudo-counts for nucleic acid *j *in each position; *a *= *a*_1 _+ *a*_2 _+ *a*_3 _+ a_4_, the total pseudo-counts in each position; *c*_*ij*_, the count of nucleic acid *j *in position *i*; and *c *= *c*_*i*1 _+ *c*_*i*2 _+ *c*_*i*3 _+ *c*_*i*4_, the total number of aligned windows, which is independent of the position *i*. The rationale behind the overall score *s *in A-GLAM is explained in detail elsewhere [17].

To initialize its annealing maximization, A-GLAM places a single window of size 3 (the default permissible minimum window size) within every sequence randomly (according to a uniform distribution), implicitly placing the windowed subsequences into a gapless multiple alignment. It then iterates the following procedure. In the procedure's first step, A-GLAM proposes a set of possible changes to the alignment. The proposal step is either a repositioning or resizing step. In a repositioning step, one sequence is selected uniformly at random; the set of proposed changes includes all possible positions for its window. In a resizing step, either the right or the left end of the alignment windows is selected; and the set of proposed changes includes expanding or contracting the corresponding end of all alignment windows by one position at a time, expansion being permitted only up to the ends of the sequences. (The resizing step has been slightly modified from its original implementation in A-GLAM, which expanded or contracted each window by a single column.) Each one of the proposed changes leads to different value of the overall score *s*. In the procedure's second step, A-GLAM then accepts one of the proposals randomly, with probability proportional to exp(*s*/*T*), where *T *is a parameter representing an annealing temperature. The temperature *T *is gradually lowered to *T *= 0, with the intent of finding the gapless multiple alignment of the windows maximizing *s*. The maximization implicitly determines the final window size. The randomness in the algorithm helps it avoid local maxima and find the global maximum of *s*. We ran the annealing algorithm within A-GLAM ten times independently. The steps (below) corresponding to E-values and post-processing were then carried out with the PSSM corresponding to the best of the ten scores *s*.

### The individual score and its E-value in the previous implementation of A-GLAM

The Gibbs sampling step produces an alignment whose overall score *s *is given by Equation (1). Consider a window of length *w *that is about to be added to A-GLAM's alignment. Let δ_*i*_(*j*) equal 1 if the window has nucleic acid *j *in position *i*, and 0 otherwise. The addition of the new window changes the overall score by

Δs=∑i=1w∑(j)δi(j){log⁡2[(cij+ajc+a)/pj]}     (2)
 MathType@MTEF@5@5@+=feaafiart1ev1aaatCvAUfKttLearuWrP9MDH5MBPbIqV92AaeXatLxBI9gBaebbnrfifHhDYfgasaacH8akY=wiFfYdH8Gipec8Eeeu0xXdbba9frFj0=OqFfea0dXdd9vqai=hGuQ8kuc9pgc9s8qqaq=dirpe0xb9q8qiLsFr0=vr0=vr0dc8meaabaqaciaacaGaaeqabaqabeGadaaakeaacqqHuoarcqWGZbWCcqGH9aqpdaaeWbqaamaaqafabaacciGae8hTdq2aaSbaaSqaaiabdMgaPbqabaaabaWaaeWaceaacqWGQbGAaiaawIcacaGLPaaaaeqaniabggHiLdaaleaacqWGPbqAcqGH9aqpcqaIXaqmaeaacqWG3bWDa0GaeyyeIuoakmaabmGabaGaemOAaOgacaGLOaGaayzkaaWaaiWabeaacyGGSbaBcqGGVbWBcqGGNbWzdaWgaaWcbaGaeGOmaidabeaakmaadmGabaWaaeWaceaadaWcaaqaaiabdogaJnaaBaaaleaacqWGPbqAcqWGQbGAaeqaaOGaey4kaSIaemyyae2aaSbaaSqaaiabdQgaQbqabaaakeaacqWGJbWycqGHRaWkcqWGHbqyaaaacaGLOaGaayzkaaGaei4la8IaemiCaa3aaSbaaSqaaiabdQgaQbqabaaakiaawUfacaGLDbaaaiaawUhacaGL9baacaWLjaGaaCzcamaabmGabaGaeGOmaidacaGLOaGaayzkaaaaaa@60D9@

The score change corresponds to scoring the new window according to a PSSM that assigns the "individual score"

si(j)=log⁡2[(cij+ajc+a)/pj]     (3)
 MathType@MTEF@5@5@+=feaafiart1ev1aaatCvAUfKttLearuWrP9MDH5MBPbIqV92AaeXatLxBI9gBaebbnrfifHhDYfgasaacH8akY=wiFfYdH8Gipec8Eeeu0xXdbba9frFj0=OqFfea0dXdd9vqai=hGuQ8kuc9pgc9s8qqaq=dirpe0xb9q8qiLsFr0=vr0=vr0dc8meaabaqaciaacaGaaeqabaqabeGadaaakeaacqWGZbWCdaWgaaWcbaGaemyAaKgabeaakmaabmGabaGaemOAaOgacaGLOaGaayzkaaGaeyypa0JagiiBaWMaei4Ba8Maei4zaC2aaSbaaSqaaiabikdaYaqabaGcdaWadiqaamaabmGabaWaaSaaaeaacqWGJbWydaWgaaWcbaGaemyAaKMaemOAaOgabeaakiabgUcaRiabdggaHnaaBaaaleaacqWGQbGAaeqaaaGcbaGaem4yamMaey4kaSIaemyyaegaaaGaayjkaiaawMcaaiabc+caViabdchaWnaaBaaaleaacqWGQbGAaeqaaaGccaGLBbGaayzxaaGaaCzcaiaaxMaadaqadiqaaiabiodaZaGaayjkaiaawMcaaaaa@4F8D@

to nucleic acid *j *in position *i*. Equation (3) represents a log-odds score for nucleic acid *j *in position *i *under an alternative hypothesis with probability (*c*_*ij *_+ *a*_*j*_)/(*c *+ *a*) and a null hypothesis with probability *p*_*ij*_. PSI-BLAST uses Equation (3) to calculate its E-values: the derivation through Equation (2) confirms the PSSM in Equation (3) as the natural choice for evaluating individual sequences.

To assign an E-value to a subsequence with a particular individual score, consider the alignment sequence containing the subsequence. Let *n *be the sequence length, and recall that *w *is the window size. If Δ*S*_*i *_denotes the quantity in Equation (2) if the final letter in the window falls at position *i *of the alignment sequence, then ΔS* = max{Δ*S*_*i *_: *i *= *w*,...,*n*} is the maximum individual score over all sequence positions *i*. We assigned an E-value to the actual value ΔS* = Δs*, as follows. Staden's method [18] yields Ρ{Δ*S*_*i *_≥ Δs*} (independent of *i*) under the null hypothesis of bases chosen independently and randomly from the frequency distribution {*p*_*j*_}. Our E-value *E *= (*n *- *w *+ 1) Ρ {Δ*S*_*i *_≥ Δs*} is therefore the expected number of sequence positions with an individual score exceeding Δs*. The factor *n *- *w *+ 1 in *E *is essentially a multiple test correction.

### The new scanning step

**Figure 1:**
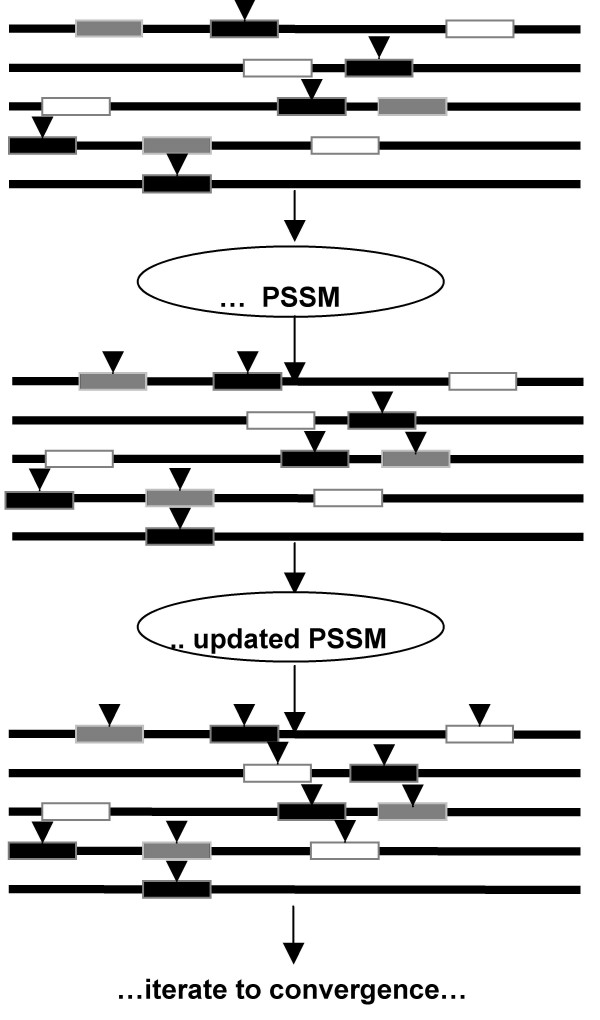
**Description of the scanning step in A-GLAM**. The figures indicate the regulatory elements with rectangular boxes. The box colors indicate the strength of the corresponding match to A-GLAM's PSSM: strong (black), moderate (striped), or weak (white). A downward arrow-tip above a box indicates that the corresponding regulatory element contributes to A-GLAM's PSSM. The top figure shows that after the Gibbs sampling step, A-GLAM predicts at most one instance of a regulatory motif per sequence. The middle figure shows that after the Gibbs sampling step, the corresponding PSSM can sweep through the sequences to predict additional instances of the regulatory motif, i.e., the instances receiving an E-value less than a (user-defined) threshold *E*_0 _now receive arrow-tips. The bottom figure shows that the previous step can be iterated, by permitting the additional motif instances to contribute to A-GLAM's PSSM, which then can sweep through the sequences once again to predict additional motif instances, indicated by new arrow-tips. The process is iterated to convergence (i.e., no new motif instances are found) or up to a (user-specified) number of iterations, whichever comes first.

Our scanning method shares some similarities with the algorithm previously developed by Neuwald et al [19]. Given a PSSM like Equation (3), the scanning step scans all sequences, assigning an E-value *E *to every subsequence of length *w*. Every subsequence with a small E-value *E *≤ *E*_0_, where *E*_0 _is some pre-assigned small threshold, contributes to the counts *c*_*ij *_in a new PSSM. The new PSSM replaces the old PSSM, and the step is repeated. The step is repeated until either: (1) no new motifs contribute to the PSSM (a condition called "convergence"); or (2) some user-specified number of iterations is attained. Figure 1 describes the method graphically. Finally, the algorithm reports the predicted motifs within each sequence, in order of increasing E-values. Analogous to PSI-BLAST, the iterative procedure usually converges, or else background motifs come to dominate the PSSM (a condition called "corruption"). Corruption indicates that a lower threshold *E*_0 _is required.

Thus, although the Gibbs sampling step in A-GLAM finds at most one regulatory element per sequence, the scanning step can rapidly locate several instances of the element in each sequence.

## Results

### Prediction performance of A-GLAM

A-GLAM's predictions of transcription factor binding sites were evaluated with reference sets containing known functional sites. Sequence logos [20] of the motifs predicted by A-GLAM were generated using WebLogo [21]. The height of a stack of letters in the logo represents the total amount of information at that position, in bits. Within each stack, the height of each letter is proportional to the nucleotide frequency at that position.

### UAS elements in histone promoters

Others have identified the SPT10 gene as a global regulator of core histone promoter activity in yeast. A recent study [22] concluded that the Spt10p transcription factor is involved in sequence-specific activation of histone genes. The protein promotes histone gene expression by binding in highly cooperative manner to paired instances of a DNA regulatory motif, UAS (upstream activating sequence). Accordingly, we tested A-GLAM with four histone promoter sequences known to contain multiple instances of the binding site for the Spt10p transcription factor. All binding sites had been experimentally verified with gel-shift assays.

**Figure 2:**
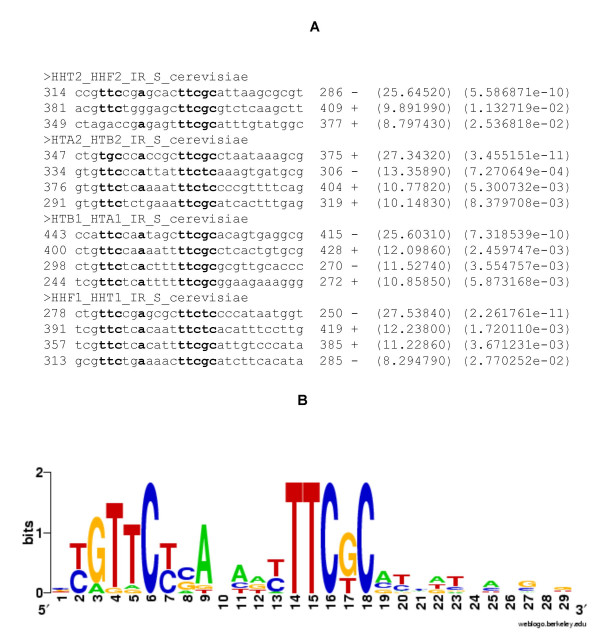
**Alignment of histone promoter sequences by A-GLAM**. Figure 2A indicates the ranking of individual elements from each sequence, based on their E-values. The six columns in each line of the alignment represent: (1) start position (2) the sequence of the predicted motif instance (3) end position (4) strand (5) individual score and (6) the corresponding E-value. The nucleotides matching the consensus are represented in bold letters. Figure 2B shows the sequence logo for the predicted motifs within the histone promoters. (The Methods section gives a brief explanation of sequence logos.) The letters at the top of the logo closely match the consensus of the histone UAS.

Of the nineteen motifs in the dataset, A-GLAM correctly identified fifteen sites without any false positives (Figure 2). A-GLAM's consensus motif of GTTCN_2_ANTTTTTCNC corresponds closely to previous results [23,24]. Previous knowledge about the consensus permits some further evaluation of A-GLAM's predictions. In the HHT2-HHF2 and HTA2-HTB2 promoters, Spt10p is known to bind to six sites. In the HHT2-HHF2 sequence, the two sites A-GLAM missed lacked the complete TTC motif, however, suggesting that Spt10p might only bind weakly there. Similarly, in the HTA2-HTB2 promoter, alignment sites contain the consensus TT/GC and TTCT/GC sites. However, the two sites A-GLAM missed lack important consensus nucleotides, again suggesting weak binding. These results suggested A-GLAM's potential to rank motifs based on binding strengths. Accordingly, we sought datasets where binding affinities had been measured experimentally.

### Operator sites in lambda phage

Many previous studies have examined the kinetics of operator binding in the promoter region of phage lambda [25,26]. Gene regulation in phage lambda is complex, and its description can be found elsewhere [27].

We extracted two sequences corresponding to adjacent promoter regions of the lambda chromosome from the RefSeq database [28]. The first promoter sequence contains the three right operator sites (OR); the second, the three left operator sites (OL). In each case, the binding sites correspond to the palindromic consensus TATCACCGCCGGTGATA [29]. Previous studies have deduced that the lambda repressor and cro compete for the operator sites, with the outcome often deciding the fate of the infected bacteria. Molecules of the repressor bound to adjacent operator sites interact and display positive cooperativity [30,31].

A-GLAM identified five out of the six experimentally verified operator sites, missing only OR2. When the five operator sites A-GLAM identified were placed in increasing order by their experimentally determined constants for dissociation with cro repressor, the E-values were also in increasing order, with the exception of OR3 (Table 1).

**Table 1:** Of six operator sites, the five identified by A-GLAM, placed in increasing order of their experimentally determined constants for dissociation with cro repressor, along with the E-values A-GLAM calculated for the individual sites.

**Operator**	**K_D_**	**E-value**
OR3	2.0 × 10^-12^	2.79 × 10^-3^
OR1	8.4 × 10^-12^	6.37 × 10^-10^
OL1	1.5 × 10^-11^	1.84 × 10^-10^
OL2	2.7 × 10^-11^	8.96 × 10^-4^
OL3	5.3 × 10^-11^	8.63 × 10^-3^

### Gal4p regulatory elements

Gal4p, encoded by the GAL4 gene, is one of the best known regulatory transcription factors in yeast. Gal4p and a complex of other proteins activate yeast galactose catabolic genes (GAL) [32]. They regulate GAL genes having multiple binding sites (*GAL1*, *GAL2*, *GAL7*, *and GAL10*) in a highly cooperative manner [33,34]. Gal4p itself binds to correctly spaced pairs of low affinity binding sites in the upstream activating sequence for GAL (UASG) [35]. Cooperativity in DNA binding causes a synergistic enhancement of Gal4p activation of transcription. Gal4p also binds to pairs of high-affinity binding sites, but binding studies involving isolated sites have shown that the corresponding UASG is only twice that of a single high affinity binding site.

**Figure 3:**
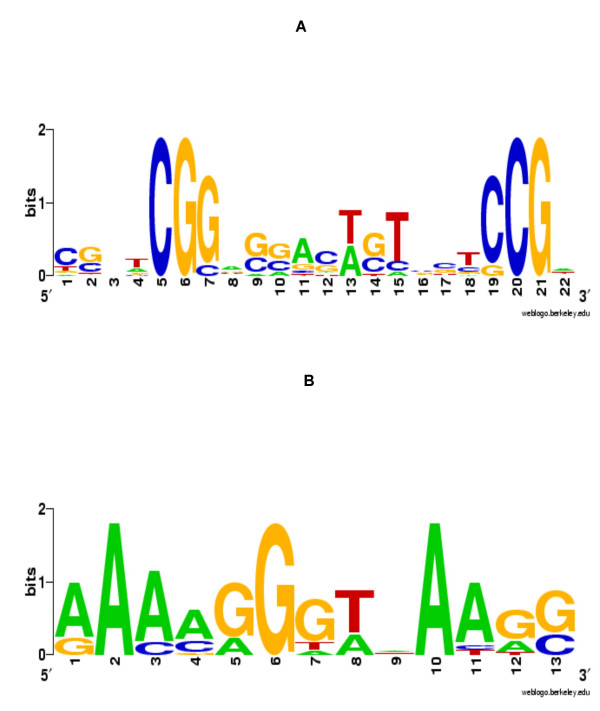
**Alignments of transcription factor binding sites**. The sequence logo generated using the motifs predicted by A-GLAM in data sets containing experimentally verified binding sites for Gal4p (Figure 3A) and Kruppel (Figure 3B).

We extracted sequences from the *Saccharomyces cerevisiae *Promoter Database for the upstream promoter regions of the six genes *GAL1*, *GAL2*, *GAL7*, *GAL10*, *GAL80 and GCY1 *[36]. The sequences contained fourteen experimentally verified Gal4p binding sites. Among the 14 binding sites, A-GLAM correctly identified 13 sites excluding one false positive. A-GLAM was run using the ZOOPS (zero or one position per sequence) mode, setting maximum motif width to 23. The consensus motif identified by A-GLAM closely matched the known consensus CGG(N_11_)CCG of the Gal4p binding site [37,38] (see Figure 3A).

### Kruppel binding targets in Drosophila melanogaster

Kruppel (Kr) encodes a zinc finger transcription factor expressed in spatially and temporally restricted patterns during Drosophila embryogenesis. We extracted genomic sequences surrounding 27 Kruppel-binding sites from 8 different genes; *Hunchback*, *tailless*, *eve*, *knirps*, *ubx*, *en*, *spalt *and *abd-b*.

A-GLAM correctly identified 12 binding sites without any false positives. The consensus produced by A-GLAM agreed well with the known consensus (see Figure 3B).

### Comparison of A-GLAM, GLAM and AlignACE

We compared A-GLAM's accuracy in identifying the binding sites with GLAM [39] and AlignACE [11], another *de novo *Gibbs sampler based algorithm. The Gibbs sampling procedure in AlignACE permits multiple occurrences of a motif in a sequence, unlike the ZOOPS model in GLAM and A-GLAM. For comparison purposes, we obtained test datasets that were used to assess various motif discovery tools in a recent contest [40]. The datasets were comprised of sequences from four different species: (1) fly; (2) human; (3) mouse; (4) yeast. Each data set contained known binding sites in the original promoter sequence (not in a random sequence). Approximately 85% of the datasets contained multiple occurrences of a binding site in at least one sequence. Hence, these datasets provided a convenient benchmark for assessment purposes. Detailed description of the datasets appear elsewhere [40]. For each tool, the accuracy of the top motif predicted on each data set was compared using the correlation coefficient

CC=(TP×TN−FP×FN)/(TP+FP)(TN+FN)(TP+FN)(TN+FP).     (4)
 MathType@MTEF@5@5@+=feaafiart1ev1aaatCvAUfKttLearuWrP9MDH5MBPbIqV92AaeXatLxBI9gBaebbnrfifHhDYfgasaacH8akY=wiFfYdH8Gipec8Eeeu0xXdbba9frFj0=OqFfea0dXdd9vqai=hGuQ8kuc9pgc9s8qqaq=dirpe0xb9q8qiLsFr0=vr0=vr0dc8meaabaqaciaacaGaaeqabaqabeGadaaakeaacqWGdbWqcqWGdbWqcqGH9aqpdaqadiqaaiabdsfaujabdcfaqjabgEna0kabdsfaujabd6eaojabgkHiTiabdAeagjabdcfaqjabgEna0kabdAeagjabd6eaobGaayjkaiaawMcaaiabc+caVmaakaaabaWaaeWaceaacqWGubavcqWGqbaucqGHRaWkcqWGgbGrcqWGqbauaiaawIcacaGLPaaadaqadiqaaiabdsfaujabd6eaojabgUcaRiabdAeagjabd6eaobGaayjkaiaawMcaamaabmGabaGaemivaqLaemiuaaLaey4kaSIaemOrayKaemOta4eacaGLOaGaayzkaaWaaeWaceaacqWGubavcqWGobGtcqGHRaWkcqWGgbGrcqWGqbauaiaawIcacaGLPaaaaSqabaGccqGGUaGlcaWLjaGaaCzcamaabmGabaGaeGinaqdacaGLOaGaayzkaaaaaa@6156@

In equation 4, *TP *(true positives) is the number of nucleotide positions common to known and predicted sites, *FN *(false negatives) is the number of nucleotide positions in known sites but outside predicted sites, *FP *(false positives) is the number of nucleotide positions in predicted sites but outside known sites and *TN *(true negatives) is the number of nucleotide positions outside both known and predicted sites.

**Figure 4:**
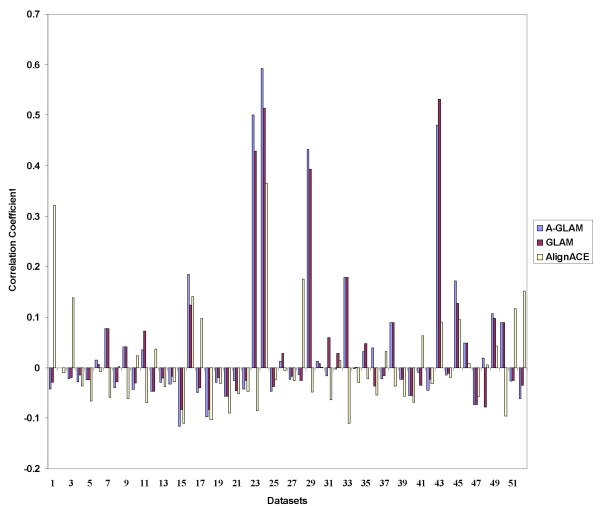
**Comparison of A-GLAM, GLAM and AlignACE**. Figure 4 shows the prediction performance of A-GLAM, GLAM, and AlignACE on 52 test data sets. (The Methods section gives a brief explanation of the data sets.) The data sets are arranged on the x-axis in the following order (1) 1–6, fly;(2) 7–18, mouse;(3) 19–26, yeast; and (4) 27–52, human. The y-axis (correlation coefficient) indicates how closely the alignments found by the 3 programs resemble the transcription factor binding site.

The comparison yielded some valuable insights into A-GLAM's performance. In general, the scanning step improved A-GLAM's ability to identify known sites on mouse and yeast datasets (see Figure 4). A-GLAM and GLAM performed poorly on fly data sets, however, worse than AlignACE. The fly datasets where AlignACE performed better contained only single sequences, however. A-GLAM and GLAM probably failed on such datasets because of their ZOOPS mode, in which the Gibbs sampler permits at most one motif occurrence per sequence. On human datasets, surprisingly, GLAM outperformed both A-GLAM and AlignACE. Moreover, the three programs often produced alignments corresponding to completely different motifs. In all such cases, A-GLAM and AlignACE identified motifs corresponding to a repeat sequence. A partial explanation follows. In the ZOOPS mode, Gibbs sampling searches for at most one motif instance in any single sequence, so the multiplicity of a repeat does not affect the Gibbs sampling step much. The best alignment after Gibbs sampling therefore might correspond to a known biological signal. Unfortunately, the large number of repeat elements in the human genome then can decoy A-GLAM in the scanning step. The multiplicity of a repeat does affect the scanning step, so after iterating sufficiently, the scanning step incorporates the repeat into the PSSM to overwhelm the original biological signal. The multiplicity of a repeat also affects the Gibbs sampling step in AlignACE, so AlignACE converges on repeats for similar reasons.

## Discussion

This paper introduces some important options into the A-GLAM computer program. Previously, after its Gibbs sampling step A-GLAM returned a gapless multiple alignment. A-GLAM assigned PSSM scores and E-values to the aligned subsequences. To avoid excessive run times, the sampling step predicted at most one regulatory element per input sequence. Now, A-GLAM uses an iterative strategy like PSI-BLAST, so the PSSM from the sampling step finds multiple instances of the regulatory motif in individual sequences. Instances with an E-value below a user-defined threshold *E*_0 _are then permitted to contribute to the PSSM, which is then updated. The PSSM-updating step is then iterated. Finally, the predicted instances of the regulatory motif are reported, by sequence in the order of increasing E-value.

Validation with regulatory elements from well characterized systems confirmed that the scanning step can identify regulatory elements rapidly and dependably, even in the presence of homotypic regulatory clusters (multiple instances of the motif in a single sequence). In comparison to Gibbs sampling for homotypic regulatory clusters, the scanning step is faster, with little loss (if any) in predictive accuracy, particularly in yeast datasets. Moreover, the E-values for predicted elements sometimes corresponded well with their experimental binding affinities (see Table 1). Further investigation of the correspondence would therefore be desirable.

The scanning step uses a threshold *E*_0 _for inclusion into A-GLAM's PSSM. The threshold *E*_0 _is critical, because it is subject to the same conflicting constraints as in PSI-BLAST. On one hand, stringent thresholds (low values of *E*_0_) can eliminate interesting instances of a motif; on the other hand, loose thresholds (high values of *E*_0_) can cause the PSSM to include too many false positives, possibly diluting the true positives to oblivion, causing "corruption". In particular, corruption can occur for subtle motifs that do not deviate much from the background nucleotide distribution. Most of our analysis used a default threshold *E*_0 _= 0.05, which is practical under most circumstances.

Our studies of A-GLAM's performance on particular datasets indicated some general conclusions about Gibbs sampling and the identification of binding sites. In a fly dataset consisting of a single sequence, GLAM's Gibbs sampling step performed poorly because the step identifies only a single binding site per sequence. The scanning step added in this article therefore identifies multiple instances of a binding site per sequence only when the dataset contains multiple sequences. The scanning step noticeably degraded predictions on human datasets, primarily because of repeats (e.g., Alu or poly-A). The degradation is likely to be a pitfall for any program able to detect homotypic regulatory clusters (e.g., either A-GLAM or AlignACE), because through sheer multiplicity, repeat elements can overwhelm a signal from homotypic binding elements. Notably, the scanning step improved A-GLAM's performance on yeast datasets, a behaviour likely to generalize to any genome containing homotypic regulatory clusters and lacking repeats. We do not specifically address the issue of interaction among binding elements for different transcription factors, a phenomenon largely confined to complex organisms. Hence, our methods are most effective in lower organisms such as yeast, fly and microbes.

Several remedies to the problem of repeats are available. First, a user can focus the A-GLAM program on a motif of interest by providing either: (1) a "seed word" contained in the motif of interest or (2) a list of "seed windows", at most one per input sequence and all of equal size. In its seed-oriented mode, A-GLAM then constrains its gapless multiple alignment to contain the user-provided seeds. Second, the repeats can be masked with standard programs, such as RepeatMasker [41]. Third, many recent studies have also suggested that a high-order background Markov model can avoid repeats and aid the detection of regulatory elements [42]. We are currently incorporating an option for a Markov background into A-GLAM.

## Conclusion

In summary, our scanning step identifies multiple elements in a single sequence with E-values. It speeds up regulatory motif discovery, by avoiding unnecessary use of the computationally expensive Gibbs sampling step, with little loss (if any) in predictive accuracy. The availability of completely sequenced genomes presents an increased demand for rapid and accurate prediction of regulatory elements. Our methods seem well adapted for this challenge.

## Availability and requirements

**Project name**: A-GLAM

**Project home page**: 

**Operating system(s)**: linux

**Programming language**: C++

**Other requirements**: gcc or other equivalent compilers

License:

**Any restrictions to use by non-academics**: NO

## Abbreviations

A-GLAM – Anchored Gapless Local Alignment of Multiple Sequences.

PSSM – Position-Specific Scoring Matrix

## Authors' contributions

KT analyzed the data and implemented the scanning step in A-GLAM; LM-R participated in the design of the study and acquired the datasets for analysis; SS provided the software for the E-value computation; DL contributed to the design of the datasets; JLS developed the mathematical theory.

## Supplementary Material

Additional file 1 The package includes: • A-GLAM source code written in C++. • A-GLAM linux executable. • Makefile Click here for file

Additional file 2 This WinZip archive contains the input test datasets used in this study. Click here for file

## References

[B1] Smale ST (2001). Core promoters: active contributors to combinatorial gene regulation. Genes Dev.

[B2] Wray GA, Hahn MW, Abouheif E, Balhoff JP, Pizer M, Rockman MV, Romano LA (2003). The evolution of transcriptional regulation in eukaryotes. Mol Biol Evol.

[B3] Ptashne M (2005). Regulation of transcription: from lambda to eukaryotes. Trends Biochem Sci.

[B4] Jensen LJ, Knudsen S (2000). Automatic discovery of regulatory patterns in promoter regions based on whole cell expression data and functional annotation. Bioinformatics.

[B5] van Helden J, Andre B, Collado-Vides J (1998). Extracting regulatory sites from the upstream region of yeast genes by computational analysis of oligonucleotide frequencies. J Mol Biol.

[B6] Sinha S, Tompa M (2000). A statistical method for finding transcription factor binding sites. Proc Int Conf Intell Syst Mol Biol.

[B7] Vanet A, Marsan L, Labigne A, Sagot MF (2000). Inferring regulatory elements from a whole genome. An analysis of Helicobacter pylori sigma(80) family of promoter signals. J Mol Biol.

[B8] Liu XS, Brutlag DL, Liu JS (2002). An algorithm for finding protein-DNA binding sites with applications to chromatin-immunoprecipitation microarray experiments. Nat Biotechnol.

[B9] Marino-Ramirez L, Spouge JL, Kanga GC, Landsman D (2004). Statistical analysis of over-represented words in human promoter sequences. Nucleic Acids Research.

[B10] Bailey TL, Elkan C (1995). Unsupervised learning of multiple motifs in biopolymers using expectation maximization. Machine Learning.

[B11] Hughes JD, Estep PW, Tavazoie S, Church GM (2000). Computational identification of cis-regulatory elements associated with groups of functionally related genes in Saccharomyces cerevisiae. J Mol Biol.

[B12] Lawrence CE, Altschul SF, Boguski MS, Liu JS, Neuwald AF, Wootton JC (1993). Detecting subtle sequence signals: a Gibbs sampling strategy for multiple alignment. Science.

[B13] Workman CT, Stormo GD (2000). ANN-Spec: a method for discovering transcription factor binding sites with improved specificity. Pac Symp Biocomput.

[B14] Frith MC, Fu Y, Yu L, Chen JF, Hansen U, Weng Z (2004). Detection of functional DNA motifs via statistical over-representation. Nucleic Acids Res.

[B15] Favorov AV, Gelfand MS, Gerasimova AV, Ravcheev DA, Mironov AA, Makeev VJ (2005). A Gibbs sampler for identification of symmetrically structured, spaced DNA motifs with improved estimation of the signal length. Bioinformatics.

[B16] Tharakaraman K, Marino-Ramirez L, Sheetlin S, Landsman D, Spouge JL (2005). Alignments anchored on genomic landmarks can aid in the identification of regulatory elements. Bioinformatics.

[B17] Frith MC, Hansen U, Spouge JL, Weng Z (2004). Finding functional sequence elements by multiple local alignment. Nucleic Acids Res.

[B18] Staden R (1989). Methods for calculating the probabilities of finding patterns in sequences. Comput Appl Biosci.

[B19] Neuwald AF, Liu JS, Lawrence CE (1995). Gibbs Motif Sampling - Detection of Bacterial Outer-Membrane Protein Repeats. Protein Sci.

[B20] Schneider TD, Stephens RM (1990). Sequence logos: a new way to display consensus sequences. Nucleic Acids Res.

[B21] Crooks GE, Hon G, Chandonia JM, Brenner SE (2004). WebLogo: a sequence logo generator. Genome Res.

[B22] Eriksson PR, Mendiratta G, McLaughlin NB, Wolfsberg TG, Marino-Ramirez L, Pompa TA, Jainerin M, Landsman D, Shen CH, Clark DJ (2005). Global regulation by the yeast Spt10 protein is mediated through chromatin structure and the histone upstream activating sequence elements. Mol Cell Biol.

[B23] Freeman KB, Karns LR, Lutz KA, Smith MM (1992). Histone H3 transcription in Saccharomyces cerevisiae is controlled by multiple cell cycle activation sites and a constitutive negative regulatory element. Mol Cell Biol.

[B24] Osley MA (1991). The regulation of histone synthesis in the cell cycle. Annu Rev Biochem.

[B25] Kim JG, Takeda Y, Matthews BW, Anderson WF (1987). Kinetic studies on Cro repressor-operator DNA interaction. J Mol Biol.

[B26] Ronald A. Albright and Brian W. Matthews (1998). How Cro and -repressor distinguish between operators: The structural basis underlying a genetic switch. Proc Natl Acad Sci U S A.

[B27] Ptashne M, Johnson AD, Pabo CO (1982). A genetic switch in a bacterial virus. Sci Am.

[B28] Maglott DR, Katz KS, Sicotte H, Pruitt KD (2000). NCBI's LocusLink and RefSeq. Nucleic Acids Res.

[B29] Sarai A, Takeda Y (1989). Lambda repressor recognizes the approximately 2-fold symmetric half-operator sequences asymmetrically. Proc Natl Acad Sci U S A.

[B30] Darling PJ, Holt JM, Ackers GK (2000). Coupled energetics of lambda cro repressor self-assembly and site-specific DNA operator binding II: cooperative interactions of cro dimers. J Mol Biol.

[B31] Johnson AD, Meyer BJ, Ptashne M (1979). Interactions between DNA-bound repressors govern regulation by the lambda phage repressor. Proc Natl Acad Sci U S A.

[B32] Keegan L, Gill G, Ptashne M (1986). Separation of DNA binding from the transcription-activating function of a eukaryotic regulatory protein. Science.

[B33] Bram RJ, Lue NF, Kornberg RD (1986). A GAL family of upstream activating sequences in yeast: roles in both induction and repression of transcription. Embo J.

[B34] Giniger E, Ptashne M (1988). Cooperative DNA binding of the yeast transcriptional activator GAL4. Proc Natl Acad Sci U S A.

[B35] Melcher K, Xu HE (2001). Gal80-Gal80 interaction on adjacent Gal4p binding sites is required for complete GAL gene repression. Embo J.

[B36] Zhu J, Zhang MQ (1999). SCPD: a promoter database of the yeast Saccharomyces cerevisiae. Bioinformatics.

[B37] Giniger E, Varnum SM, Ptashne M (1985). Specific DNA binding of GAL4, a positive regulatory protein of yeast. Cell.

[B38] Liang SD, Marmorstein R, Harrison SC, Ptashine M (1996). DNA sequence preferences of GAL4 and PPR1: how a subset of Zn2 Cys6 binuclear cluster proteins recognizes DNA. Mol Cell Biol.

[B39] Tompa M, Li N, Bailey TL, Church GM, De Moor B, Eskin E, Favorov AV, Frith MC, Fu Y, Kent WJ, Makeev VJ, Mironov AA, Noble WS, Pavesi G, Pesole G, Regnier M, Simonis N, Sinha S, Thijs G, van Helden J, Vandenbogaert M, Weng Z, Workman C, Ye C, Zhu Z (2005). Assessing computational tools for the discovery of transcription factor binding sites. Nat Biotechnol.

[B40] Smit A HRGP (1996). RepeatMasker Open-3.0.. http://www.repeatmasker.org/.

[B41] Thijs G, Lescot M, Marchal K, Rombauts S, De Moor B, Rouze P, Moreau Y (2001). A higher-order background model improves the detection of promoter regulatory elements by Gibbs sampling. Bioinformatics.

